# A Novel Electronic Data Collection System for Large-Scale Surveys of Neglected Tropical Diseases

**DOI:** 10.1371/journal.pone.0074570

**Published:** 2013-09-16

**Authors:** Jonathan D. King, Joy Buolamwini, Elizabeth A. Cromwell, Andrew Panfel, Tesfaye Teferi, Mulat Zerihun, Berhanu Melak, Jessica Watson, Zerihun Tadesse, Danielle Vienneau, Jeremiah Ngondi, Jürg Utzinger, Peter Odermatt, Paul M. Emerson

**Affiliations:** 1 The Carter Center, Atlanta, Georgia, United States of America; 2 Department of Epidemiology and Public Health, Swiss Tropical and Public Health Institute, Basel, Switzerland; 3 University of Basel, Basel, Switzerland; 4 Department of Computer Science, Georgia Institute of Technology, Atlanta, United States of America; 5 The Carter Center, Addis Ababa, Ethiopia; 6 Department of Public Health and Primary Care, Institute of Public Health, University of Cambridge, Cambridge, United Kingdom; Kenya Medical Research Institute - Wellcome Trust Research Programme, Kenya

## Abstract

**Background:**

Large cross-sectional household surveys are common for measuring indicators of neglected tropical disease control programs. As an alternative to standard paper-based data collection, we utilized novel paperless technology to collect data electronically from over 12,000 households in Ethiopia.

**Methodology:**

We conducted a needs assessment to design an Android-based electronic data collection and management system. We then evaluated the system by reporting results of a pilot trial and from comparisons of two, large-scale surveys; one with traditional paper questionnaires and the other with tablet computers, including accuracy, person-time days, and costs incurred.

**Principle Findings:**

The electronic data collection system met core functions in household surveys and overcame constraints identified in the needs assessment. Pilot data recorders took 264 (standard deviation (SD) 152 sec) and 260 sec (SD 122 sec) per person registered to complete household surveys using paper and tablets, respectively (*P* = 0.77). Data recorders felt a lack of connection with the interviewee during the first days using electronic devices, but preferred to collect data electronically in future surveys. Electronic data collection saved time by giving results immediately, obviating the need for double data entry and cross-correcting. The proportion of identified data entry errors in disease classification did not differ between the two data collection methods. Geographic coordinates collected using the tablets were more accurate than coordinates transcribed on a paper form. Costs of the equipment required for electronic data collection was approximately the same cost incurred for data entry of questionnaires, whereas repeated use of the electronic equipment may increase cost savings.

**Conclusions/Significance:**

Conducting a needs assessment and pilot testing allowed the design to specifically match the functionality required for surveys. Electronic data collection using an Android-based technology was suitable for a large-scale health survey, saved time, provided more accurate geo-coordinates, and was preferred by recorders over standard paper-based questionnaires.

## Introduction

Large-scale national and sub-national surveys are needed to map the distribution of a health condition, establish baseline prevalence and incidence data for planning, and monitoring the impact of neglected tropical disease control interventions [1]–[5]. These surveys often involve collection of a combination of geospatial and disease prevalence data from multiple households within multiple communities across many different regions of a country. Sample sizes vary according to program-specific methodologies, but typically thousands of individual, household, school, and community records are obtained on paper forms [6]–[11]. Paper forms must be printed, transported to the field, distributed, filled in, collected, organized, collated, and kept secure prior to data entry. Once data have been collected, forms must then be entered manually into computer databases. To ensure quality of stored data, the forms should be entered twice by separate data entry clerks and then compared to remedy discordant entries against the original hard copy forms. Finally, in good practice, the paper forms must be stored securely for a minimum time period (no less than 2 or 5 years after publication) according to national and international regulations [12], [13].

As neglected tropical disease control and elimination programs seek to assess impact of interventions, large sample sizes are required to have the power to determine low levels of disease [14]. For example, while 10 communities may be acceptable for estimating the need to initiate control interventions to over a million population in a “super district” suspected hyperendemic for trachoma, 10 or more clusters per 50,000 population in a sub-district are required to document low-level disease before stopping interventions [15]. Applying sub-district-level surveys in only two zones (population of 3.8 million) of the Amhara National Regional state in Ethiopia required collecting data from over 21,000 households in 714 communities [16]. A 4-page paper survey tool was employed and administered to each household. Needless to say that paper-based surveys at this magnitude require significant time, financial and human resources, and physical storage space.

Electronic data collection has been proposed as a solution to the challenges posed by paper-based surveys and several advantages have been discussed [17]. Devices such as mobile phones, using short message service (SMS), have been effectively used to manage drug-stock in rural health facilities, push health communication messages to target populations and for disease surveillance [18]–[20]. However, the amount and complexity of the data collected and sent with SMS is limited [21]. Personal data assistants (PDAs) allow the capture of more complex data and efficient use of PDAs has been documented in disease surveillance and clinical research, as well as national surveys [22]–[25]. With the development of the Android (Google Inc.) platform, applications such as Open Data Kit (ODK; www.opendatakit.org) and EpiCollect (www.epicollect.net) have broadened the options in mobile data collection in public health to so-called ‘smart’ devices (primarily touch-screen mobile devices, such as smart phones and tablet computers) [26], [27]. Many of these devices offer the additional advantage of having built-in global positioning systems (GPS) to automatically capture geographic coordinates from external GPS devices, as opposed to transcribing coordinates to paper-surveys from external GPS devices, thus minimizing transcription errors in the field.

The purpose of this study was to evaluate the use of a novel electronic data collection system for use on tablet computers operating on the Android platform, and to determine whether this system was feasible and as effective as standard paper-based forms in collecting data in large-scale household surveys in a remote area of Ethiopia with poor infrastructure. Additionally, an effort was made to estimate person-time days and cost incurred by the two approaches of data collection.

## Materials and Methods

### Ethics Statement

The pilot study reported here was integrated into a training exercise for the trachoma impact assessment surveys approved under Institutional Review Board protocol 079–2006 of Emory University and by the Amhara National Regional Health Bureau ethical review committee. Data obtained from these surveys and used for the current comparison are the property of the Amhara National Regional State Health Bureau and The Federal Ministry of Health of Ethiopia and made accessible to The Carter Center under a memorandum of understanding, but are not publically available. Details of the ethical consideration for these surveys and training have been explained elsewhere [16]. Participants involved in needs assessment discussions were aware of the intent to publish from the outset of the discussion, are authors on the present paper, and have consented to the contents of this paper.

### Study Preparation

We consulted freelance volunteers (senior computer science undergraduates) with experience, expertise, and interest in seeking to apply their technical skills in a philanthropic project. In a first step, we conducted a needs assessment prior to proposing an electronic data collection system aimed at improving efficiency in large-scale household surveys. This involved group discussions with the volunteer computer scientists, epidemiologists, and public health program officers with extensive experience in cross-sectional household surveys to identify core, functional needs of both hardware and software when implementing a survey, and identifying potential constraints of electronic data collection under typical field conditions in resource-constrained settings. Next, we piloted a proposed, novel electronic data collection system in a setting where such a system would be deployed to further refine the design and provide developers first-hand experience of real constraints faced in the field. This pilot involved the following activities: training data recorders experienced in paper-based surveys to use an Android device and electronic questionnaire; recording the time required for data collection using the same household questionnaire by either paper and pencil, or electronically on a tablet computer; and finally, documenting the perceptions of the data recorders about using the electronic data collection system. The hardware and software used for electronic data collection in the study activities are described in Table S1.

### Training and Study Implementation

The pilot survey team consisted of eight members who had previously been deployed in a large-scale trachoma survey in Ethiopia. The same survey tool (paper questionnaire, see Document S2) that was used by the teams in the large-scale survey was designed electronically and loaded onto tablet computers.

The data recorders were trained for one day in a classroom on how to operate the tablet computer and collect data using the Android application, including capture of geographic coordinates. This was followed by one day of practice in a nearby community whereby the eight members were split into two teams of four (two data recorders and two trachoma examiners) and adhered to the same protocol as of previous surveys. At each household, one data recorder administered the questionnaire and collected data using the tablet, while the other recorded data on the paper questionnaire. The recorders took turns using the tablet and alternating lead interview roles at each household for a total of 20 households. The next two days the same process was repeated in two separate communities. Without the data recorders’ knowledge, the team supervisors recorded the time required for the data recorder to implement the survey at each house and whether it was administered by paper or tablet. On each day of applied training, the participants’ perceptions on using either paper-questionnaires or electronic questionnaires were documented through focus group discussions (FGDs). Using a grounded theory approach, these discussions were semi-structured around a core set of questions (Document S3) and new themes identified in one FGD were explored in subsequent FGDs until saturation of themes was reached [28]. Additionally, the participants were given the same questions on individual questionnaires and were asked to share any comments they felt were not adequately discussed or did not want to openly share in the discussion. Two observers entered separately the responses and any additional comments provided by the participants which were compared for consistency. The discussion notes were reviewed by the coauthors and emerging common themes extracted as has been done in other programs [29]. The findings from the FGDs were used to revise the survey instrument and functionality of the data collection program, and were re-tested among the study participants.

### Data Comparison

The electronic data collection system was further refined and then implemented in a large-scale trachoma prevalence survey using the same sampling methodology (i.e., population-based, multi-stage probability sampling) and questionnaire as implemented 7 months prior in a neighboring zone of the same region of Ethiopia. The sampling methodology and results of these surveys are described in detail elsewhere [16]. Of note, the cluster size was increased in the survey using electronic data collection by randomly selecting one segment of 40 rather than 30 households as done in the previous survey in order to meet the target sample size for estimating prevalence of trachoma among children aged 1–9 years.

The first survey collected data on standard paper-based questionnaires and the other utilized the modified electronic data collection system both using survey tool (Document S1). The survey tool consisted of one interview per household to obtain household characteristics, head of household demographics, knowledge of trachoma, knowledge of prevention measures, reported behavior of face washing and household indicators of water, sanitation, and hygiene. Nested within the household interview, data was collected from all individual household residents including demographics, reported school attendance among children and participation in antibiotic mass distribution, and clinical examination for trachoma, hence creating the hierarchical structure of parent (household) to child (individual resident). An additional questionnaire was employed for one randomly selected school-aged child per household by branching to a separate electronic form linked to the parent and child data with a unique identification number. This additional questionnaire was not used in the survey employing paper questionnaires and thus is not compared in the analysis.

### Analysis

We assessed the raw data sets from the two large-scale trachoma impact assessment surveys conducted 7 months apart. We compared the difference in frequencies of survey refusals and identified data entry errors using a Χ^2^ test and *t*-test corrected for the survey design where appropriate. We focused on data entry errors that could have the most impact on disease prevalence estimates: number of blank fields (i.e., missing data where data should have been recorded according to protocol); age and sex of participants; availability for examination; incorrect unique identifying number; a blank field in the classification of trachoma clinical signs; or an impossible combination of clinical signs (e.g., no signs and clinical signs recorded for the same eye).

Geographic coordinates as recorded by the GPS were collected for every household, in each community cluster, for both surveys. Using the recorded household coordinates, we calculated the median location for each community surveyed to serve as the cluster centroid. We then computed the distance between the coordinates for each household to the cluster centroid, and compared the two surveys with a *t*-test. The Euclidian distance was calculated using the Haversine formula [30], and cross-checked in ArcGIS version10 (ESRI; Redlands, CA, United States of America) by re-projecting the latitude and longitude (in decimal degree) into Northing and Eastings (in meters, UTM Zone 37). We also compared the frequency of obvious outlying household coordinates, defined as ≥4 km away from the cluster centroid. Finally, we mapped all households linked to their cluster centroid to visually assess the differences in accuracy of each data collection method. Statistical tests were conducted in STATA version 12.0 (Stata Corp.; College Station, TX, United States of America).

To estimate costs associated with paper-based data collection, we used 10.9% of a median cost per cluster of US$ 311 (inter-quartile range [IQR]: US$ 119–393) estimated from a previous study of trachoma prevalence surveys in 165 districts across eight countries [31]. This was the overall proportion of total costs due to data entry of paper questionnaires. An additional 1.5% of total cluster cost was assumed to cover the cost of the paper and printing for questionnaires. For a conservative estimate of the cost of electronic data collection, we simply took the sum of equipment costs for the tablet computer and accessories, assuming a one-time use.

## Results

### Design of Electronic Data Collection System

**Figure 1:**
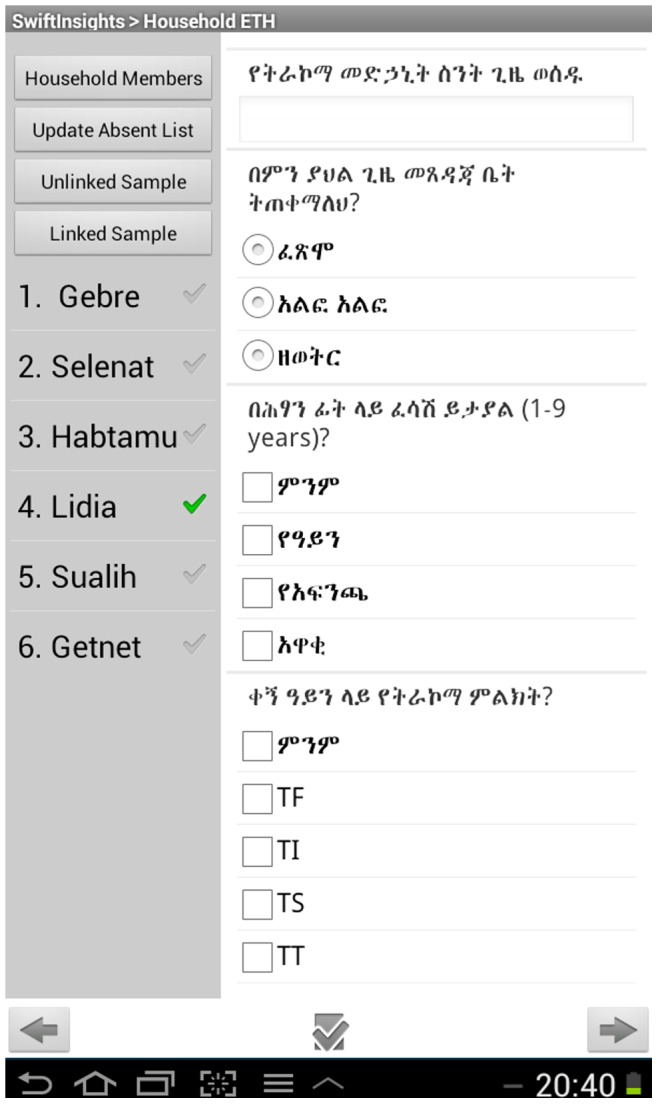
Example screen shot: looping fields for members grouped within a household record. As seen in a novel Android application for collecting data in household surveys.

The summary of the findings of the needs assessment and developed solutions are listed in Table 1. In brief, the proposed electronic data collection system incorporated a desktop user-friendly interface, which readily allowed survey planners to design, modify, and update electronic questionnaires, without the need for Internet connection, in an intuitive drag-and-drop form builder. Additionally, the Android application to collect data was able to capture recurring data from individuals within a data record collected from the household. Unique identification numbers were generated based on survey preferences set on the tablet in the home screen of the application and from minimal input of the data recorder. Data fields for each individual enumerated in a household record were arranged alongside the list of enumerated individuals, which allowed flexibility of alternating between individuals as a survey team encountered each one for examination (Figure 1). From this same display, the random selection functions were accessible, allowing random selection of an eligible individual enumerated in the household record for additional assessment (e.g., submission of stool sample for the diagnosis of intestinal parasites). Eligibility was defined as set by the user in survey preferences accessed from the home screen. To manage the survey, the user interface allowed the distribution of created forms, uploading, and appending collected data through USB connection without Internet connection.

**Table 1:** Needed functionality of electronic data collection in household surveys and the solutions implemented.

	Description of need	Final solutions implemented
**Software** Creation	▪ Simple design of new surveys▪ Update of existing surveys▪ Display multiple languages	▪ No Internet connection required for design, drag-and-drop survey builder▪ Create and save templates for fast production of new surveys▪ Entry of multiple translations in form builder▪ Export labels for faster, bulk translation
Collection	▪ Simple data entry▪ Accommodate skip patterns▪ Generate unique identification numbers for each householdand individual▪ Maintain parent-child relationships of household-level and individual-level data▪ Generate random samples of entered records▪ Track external specimens▪ Minimize errors▪ Input text in multiple languages	Android application with base functionality of Open Data Kit plus:▪ Ability to generate relational databases so that data entered once applies to all related records▪ User defined survey preferences▪ Generation of unique record identification▪ Select enumerated residents randomly▪ Save listing of absent persons for ease of review and completion▪ Side-by-side view of enumerated residents and repeating data fields (Figure 1)▪ Capture input from internal or external GPS and camera▪ Language specific keyboards
Management	▪ Efficient distribution of survey forms▪ Minimal risk of data loss▪ Append data from multiple devices▪ Convert data to a generic file format for broad compatibility▪ Reduce time to data availability	▪ Data written to external storage on device▪ Without Internet or mobile network connection, from a local desktop user-friendly interface:1. Distribute forms2. Upload and append collected data3. Convert data to useable format
**Hardware** Durability	▪ Withstand heat, cold, moisture, and dust▪ Battery life for at least 1 working day▪ Ease of recharging	▪ Android tablet computer 7″ display▪ Internal battery minimum capacity 6–8 hr▪ Protective case▪ Portable external battery pack▪ AC and DC to multiple USB plugs for charging
Capability	▪ Capacitive touch screen▪ Visible display in bright sunlight▪ Collection of geographic coordinates▪ Camera for scanning barcodes▪ Recoverable data	▪ Tablet computer with 3.5 mega pixel camera▪ Auto-brightness display setting▪ Internal GPS▪ Removable, external micro SD cards

**Figure 2:**
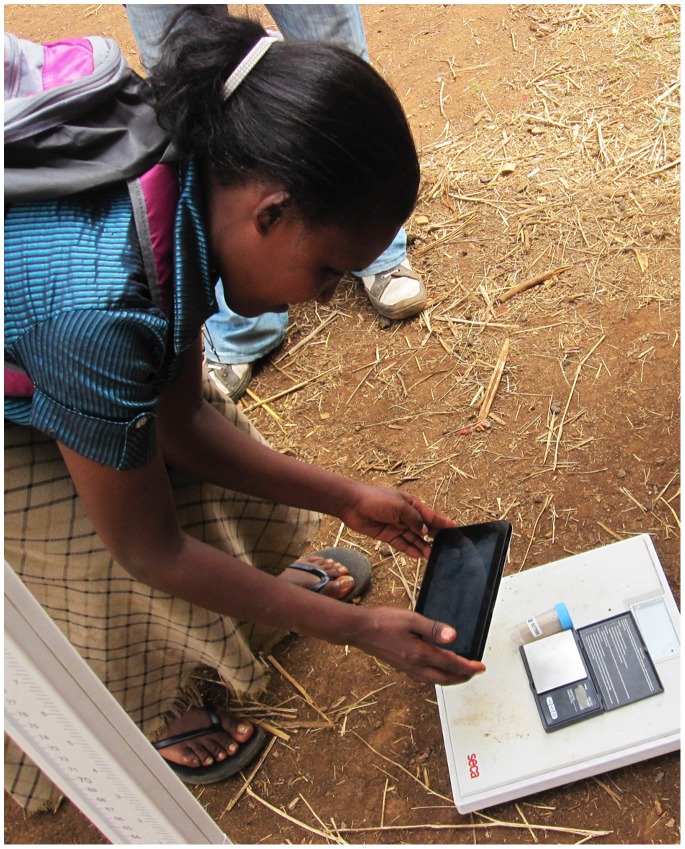
Capturing the identification number from a barcode-labeled stool specimen. As conducted during an integrated survey of neglected tropical diseases in Amhara National Regional state, Ethiopia in 2011.

With regards to hardware (Box 1), during the pilot testing, we used a tablet computer with a 7-inch resistive touch screen display with exchangeable batteries. The resistive screen was tough, but not sensitive to normal touch and the external batteries proved problematic to exchange on-the-go and keep charged. Hence, for the full deployment, we used a tablet with 7-inch capacitive touch display enabling softer touches to the screen and a more responsive user experience. The deployed tablet had an internal battery providing 6–8 hours of use, which could then be recharged through a USB connection to AC/DC or external battery pack charging units. An internal GPS allowed the direct capture of geographic coordinates. To minimize errors in linking results to household and individual records and maintain privacy of survey volunteers, a camera with autofocus allowed use of an application (Barcode Scanner 4.3.1; see http://code.google.com/p/zxing) to capture of random identification numbers from 0.25″×0.25″ QR codes on 1.18″x0.5″labels used to uniquely link external specimens (in this case, stool specimens; see Figure 2). Data were stored on an external micro SD card to reduce risk of data loss.

### Results from Pilot Investigation

A total of 40 households were surveyed over two days in two separate communities during pilot testing. There was no difference in the time required to collect data between the paper-based and electronic method over the 2-day observation period (Table 2). The time taken to enter data when the survey was administered with tablets was 48 sec per person more on the first observed day (day 2) and 54 sec per person less on the second observed day (day 3) compared to paper surveys. These differences, however, were not statistically significant (*P* = 0.20 and *P* = 0.50, respectively).

**Table 2:** Time to complete paper-based and Android-based electronic questionnaires during a pilot trial in Ethiopia 2011.

	Paper*	Tablet*	H_0_: Paper = Tablet**
**Day 2**			
Total number of households surveyed	10	10	
Mean number of residents per household	4.9 (1.8)	4.5 (2.9)	
Mean time (sec) to enter data per person registered	268 (101)	320 (119)	z = −1.29 *P* = 0.20
**Day 3**			
Total number households surveyed	10	10	
Mean number residents per household	3.8 (1.2)	3.9 (1.2)	
Mean time (sec) to enter data per person registered	260 (197)	201 (97)	z = 0.68 *P* = 0.50
**Combined 2-Day Results**			
Mean time (sec) to enter data per person registered	264 (152.4)	260 (122)	z = −0.30 *P* = 0.77

* SD- standard deviation.

** Wilcoxon rank-sum test.

After the field work, recorders shared their perceptions concerning electronic data collection with key findings summarized in Table 3. Recorders felt the paper survey took less time to complete, but they enjoyed learning the new technology and preferred to collect data electronically in future surveys. Recorders expressed a lack of connection to the respondent when first learning to use the data collection device due to less eye-to-eye contact while administering the questionnaire. The training curriculum was modified to address attentiveness and connection to the respondents in future surveys. The greatest concern about using electronic data collection was the ability to keep the electronic device charged under field conditions. We also included modules in future survey trainings to address power management and common data collection mistakes or difficulties as reported by the pilot data recorders (Table 3).

**Table 3:** Data recorders’ perceptions of electronic data collection post 3-day pilot trial in Ethiopia.

Aspect explored	Summarized perceptions
**Time**	▪ Paper questionnaire took less time to complete than the electronic questionnaire
**Preparation**	▪ No printing, sorting, stapling, and labeling with unique numbers is required with electronic data collection
**Transporting**	▪ Tablet computers were portable, lighter, and less bulky than paper
**Communication with respondents**	▪ Less eye-to-eye contact with respondent, but was less of a problem once familiar with the tablet computer
**Recording data**	▪ Transcribing GPS coordinates onto paper forms was a difficult task and the direct capture of GPS coordinates via the tablet was preferred▪ Writing district, village, and community names on a paper form for every household was tedious▪ No writing necessary for electronic data collection▪ Recorders must be attentive to skip patterns on a paper form, but the skip patterns were automatic on the electronic form▪ Entering text, moving the cursor, and editing text fields were most challenging tasks using the tablet computers▪ Accidental selections on single select (i.e., yes or no) questions when the question did not apply could not be de-selected only switched to either option▪ Mistakes on paper forms can be erased and corrected▪ More difficult to return to a completed electronic form and add information than a paper form (i.e., an absent person presents for examination after the survey team has moved to a new household)
**Data management**	▪ Risk of losing the data was greater for tablets than for paper forms because paper is tangible▪ Paper forms are difficult to keep clean, dry, and in order
**Training**	▪ Ability to use tablets may be enhanced by experience in using computers▪ Data recorders should become familiar with questionnaires first before using tablets▪ Power management must be covered
**General concerns**	▪ Keeping device charged where there is no access to electricity
**General preferences**	▪ Enjoyed learning new technology▪ Questions on the electronic form and entry of data in *Amharic* (native language) are preferred▪ Use electronic data collection rather than paper questionnaires in future trachoma surveys

Data loss with paper surveys was perceived less risky than with electronic data collection since the paper questionnaires were tangible, enabling the immediate review, identification, and correction of mistakes. Paper surveys were also perceived to be easier to manipulate, to add or change data, including an absent household member who was later encountered by the survey team. To address these concerns the Android application was modified to allow identification of absent persons in a household, and aggregate these people in an absent list that facilitates finding the correct household record and completing the necessary fields of the presenting absent person.

### Results from Large-Scale Surveys

Outcomes from the paper-based and electronic data collection in separate, large-scale surveys utilizing the same sampling methodology and questionnaire are presented in Table 4. The surveys were equivalent in scope and scale. Refusals to participate were rare, but significantly more common among household residents when using the electronic device (0.8%, 95% confidence interval (CI), 0.7–1.0%) compared to paper-based surveys (0.3%, 95% CI 0.2–0.4%) (*P*<0.01). The number of empty entries for the fields age, sex, and availability of enumerated household residents was fewer with electronic data collection than paper-based collection by 0.2% (*P* = 0.01). There were fewer errors identified in the unique identifying numbers of each household in the electronically collected data set (1.8%) than the paper-based data set (2.3%) (*P* = 0.09). There was no difference in the amount of errors made when recording the trachoma clinical diagnosis between the two data collection approaches (0.2% *vs.* 0.2%, *P* = 0.26).

**Table 4:** Data comparison of paper-based and electronic data collection from two large-scale, cluster surveys in Ethiopia.

Indicator compared	Paper-based data collection	Electronic data collection	χ^2^ or t-test (p-value)
**Sample**			
Clusters	360	354	NA
Households surveyed	9,263	12,064	NA
Individuals enumerated	38,851	50,858	NA
Individuals examined	33,800	38,652	NA
**Refusals**			
Individual-level	0.3% (N = 38,852)	0.8% (N = 50,884)	27.96 (*P*<0.01)
**Identified data entry errors**			
% Individuals enumerated with at least 1 blank field incensus record (age, sex, availability)	1.7% (N = 38,851)	1.5% (N = 50,858)	6.61 (p = 0.01)
% households with incorrect unique identifying number	2.3% (N = 9,433)	1.8% (N = 12,112)	6.83 (*P* = 0.01)
Disease classification	0.2% (N = 33,800)	0.2% (N = 38,652)	1.28 (*P* = 0.26)
**Geographic coordinates**			
Blank entries	0.6%(N = 9,263)	1.1% (N = 12,064)	12.14 (*P*<0.01)
Outlying entries^‡^	1.4%	0.6%	38.92 (*P*<0.01)
Mean household distance in meters to cluster centroid (SE)	687 (81)	288 (7)	t = −5.53 (*P*<0.01)

‡ Defined as recorded households with coordinates more than 4 km from cluster centroid, or more than 1,000 m elevation from median elevation of the cluster.

NA, not applicable.

**Figure 3:**
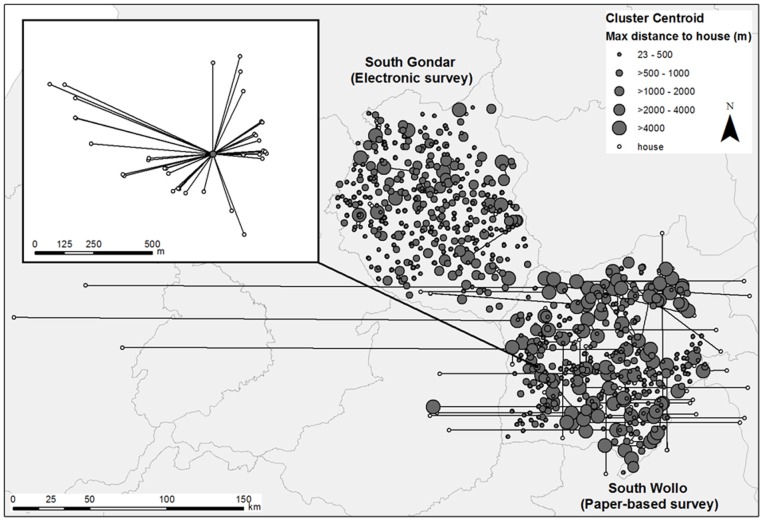
Distance between the recorded location of a surveyed household and the cluster centroid. Households surveyed in trachoma impact assessments in South Wollo (paper-based questionnaire 2010) and South Gondar (electronic data collection 2011), Ethiopia.

When comparing the capture of geographic coordinates, 0.5% more empty fields were observed in the data collected with tablets than in the paper-based data (*P*<0.01). Outliers, defined as household coordinates ≥4 km from the median geographic coordinate in the surveyed cluster or more than 1,000 m elevation from median elevation in the cluster, were more common in paper-based collection than electronic collection (1.4% *vs*. 0.6%, *P*<0.01). The mean distance from a household in a cluster to the cluster centroid was 400 m greater in the survey where coordinates were transcribed to paper questionnaires compared to electronic survey application (*P*<0.01). Variability of this distance is seen in Figure 3, where each point represents a household and each solid circle represents the cluster centroid. Each household is connected by a line to its cluster centroid circle. The displayed size of the circle is proportional to the maximum distance between a household and the centroid in that cluster.

**Figure 4:**
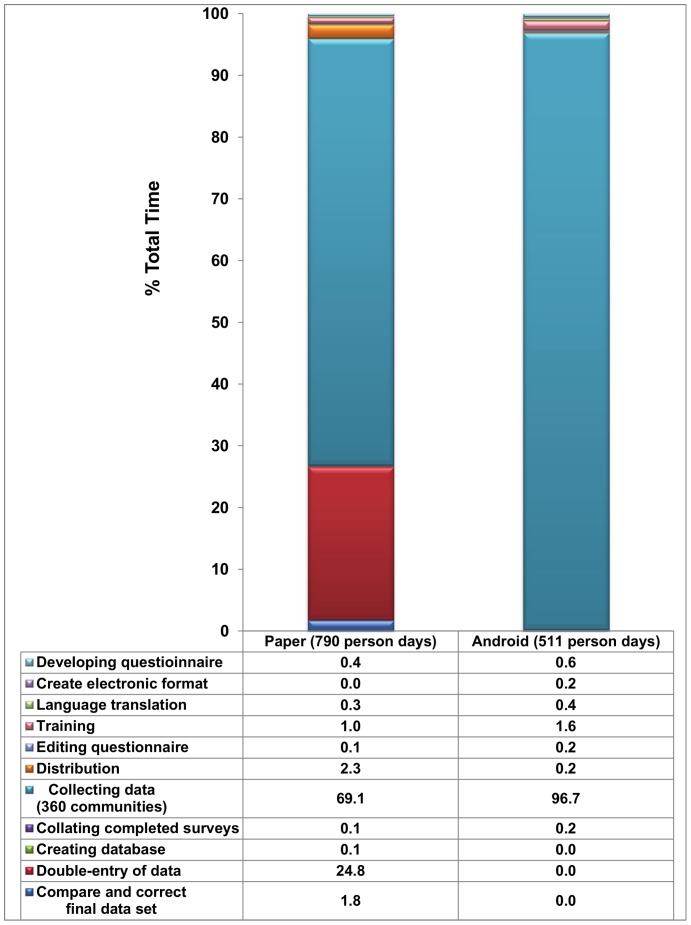
Proportion of total time (person days) required to complete survey activities by collection method. Time as implemented using paper-based questionnaire and Android-based electronic form in two large-scale (360 clusters each) trachoma impact assessments in Ethiopia 2010 and 2011.

The total time taken to prepare, implement, and process the data was 511 and 790 person-days for electronic and paper-based data collection, respectively. The proportion of time taken to complete activities involved with data collection is presented in Figure 4 for both surveys. The two survey methods required approximately the same amount of time to develop the questionnaire, obtain translations and edit. An additional person day was required to convert the paper-questionnaire to an electronic platform. For either method, the length of training was approximately one full week. The paper-based questionnaires (over 9,000 in total) took 18 person-days of preparation to print, collate, staple, and distribute prior to deploying teams for actual fieldwork. Preparing the electronic survey and loading to 20 electronic collection devices took less than one person-day. Collecting data in the field in South Wollo (paper-based survey) took 21 survey teams 26 days to complete, while in South Gondar (electronic survey) it took 13 teams 38 days; the latter, 52 person-days fewer. Including the time required for survey teams to access the selected clusters, one cluster was completed every one and half days per team in both surveys. Upon completion of field work, data collected electronically was uploaded to the survey coordinator’s computer, appended, and converted to a usable data set in less than one day using the desktop interface. Completed paper questionnaires were collected from all teams, transported back to a central office where 14 data entry clerks working 8–10 hours per day for 14 days double-entered the data into separate databases. An additional 5 days was required to compare the duplicate data sets for discordant records, find the respective hard-copy questionnaires, and identify the correct entry before a final data set was available. Together, these data entry and correction activities accounted for 26.6% of the total time spent on the paper-based survey.

### Cost Estimates

Costs associated with paper-based data collection in the compared survey were US$ 13,883, which included an estimated cost of US$ 1,679 for paper and printing of questionnaires plus US$ 12,204 for entering the data from questionnaires twice by separate data entry clerks into a database. The incremental survey costs associated with electronic data collection were US$ 10,320, which included 24 tablet computers (US$ 299 per piece), carry cases (US$ 15), micro SD memory card for external data storage (US$ 12), two external batteries for charging the tablet in absence of electricity (US$ 40 each), AC-USB adapter for charging with electricity (US$ 12), and DC-USB adapter for charging in a vehicle (US$ 12). These equipment costs assume a single use, which, more realistically, may be used for multiple surveys thus lowering incremental costs. Additionally, four of 24 tablet computers were not needed and never deployed during the survey.

## Discussion

In this study, electronic data collection was at least as accurate as data collected using conventional paper-based questionnaires. Importantly, the novel electronic data collection system was less time consuming and our preliminary cost evaluation suggests that it is less costly. There was no evidence of differences in the amount of data entry errors identified by the two data collection methodologies. However, the differences in accuracy and precision observed between the captured geographic locations of surveyed households were significant, which, in turn, could affect subsequent spatially explicit data analysis. The accuracy of data entry using the electronic system could be further enhanced with additional logic statements and by blocking impossible combinations of entered data. The costs of the equipment required for electronic data collection was approximately the same cost required for data entry of paper-based questionnaires in a single survey, which was a conservative estimate. Indeed, use of the electronic data collection equipment in additional surveys would further reduce costs.

In the pilot study, there were no differences in time to completion of the household surveys between the methods, which was also reported in a comparison of smartphone administrated interviews among attendees of maternal health clinics in the People’s Republic of China [32]. In comparing the two large-scale surveys, more households and individuals were examined in the electronic survey because of a modification in protocol to select more households per cluster. Yet, even with the increased work load, teams collected more data in less time, as expressed in person-days to collect data using electronic capture. A total of 265 person-days were gained when using the electronic data collection system. The majority of time saved came from obviating the need for post-field collection data entry and translated into having the final data set available for analysis nearly one month sooner. Time saved is invaluable in program settings, as it allows for immediate reporting of results to decision makers within the health system and creates lead time for preparation of needed interventions or importation of commodities such as donated drugs. Because impact surveys where data is collected electronically requires less time than conventional paper-based methods, the flexibility of when the survey is conducted within the program schedule is increased. This is a critical advantage in neglected tropical disease control programs due to the tight and often complex calendar of planning annual mass drug administration campaigns and other community-based interventions [33]–[36].

Pilot testing was crucial and identified additional flexibility needed in the electronic data collection system and further insight into the type of hardware required. It also introduced us to the perspectives of the experienced data recorders from previous paper-based data collection, which raised important issues that were addressed in the final system and provided insight to the training curriculum used in the large-scale deployment. We tailored the selected Android device to fit the needs of the survey and field conditions, which required extended battery life, internal GPS, touch screen, no stylus or external keyboard, and automatic adjustment of display brightness.

We applied a novel electronic data collection system in this study that did not require short-cuts or redesigning the survey forms to absolute minimum requirements due to limited functionality, as observed in use of PDA and SMS-based systems [37], [38]. The software was designed to fit the need, rather than the survey designed to fit the capabilities of the electronic system. The Android application for collecting data was designed to mimic the protocol of the survey team and offer sufficient flexibility to match the dynamics of interactions with household residents, which may have been contributing factors to explain why experienced, paper-based data recorders wished to use electronic data collection in the future. For example, household information needed entry only once for each individual in a household. Each household resident could be registered regardless of availability and data collection could occur for each resident in any order simply by selecting the person in the side-by-side view and entering the individual’s trachoma signs (or other information). This system also provides the flexibility to have multiple translations and input data in multiple languages, like Amharic, which was a cited preference of the pilot data collectors.

The desktop user interface allowed a multi-level survey prepared first on paper, to be transferred to an electronic form and applied in an application on any Android device within a day, without requiring mobile phone or Internet connections. Forms can be uploaded to survey devices for deployment within minutes while simultaneously downloading collected data held on the device. Data are protected securely on the device using a log-in code or pattern, stored on an external disc housed within the collection device minimizing risk of data loss. We were not able to compare the frequency and type of lost data between the paper-based and electronic surveys, and therefore cannot address the perceptions from data recorders of the greater risk of data loss with electronic collection. We had three out of 24 devices temporarily power-off and fail to reboot, though data was recovered by removing and downloading data stored on the external SD card. The devices were set aside, but most importantly, all three rebooted after a 48-hour re-charge and remained functional even though not needed for the rest of the survey. Data were downloaded to the password-protected laptops of supervisors at least every second day, which further minimized risk of data loss should the device be stolen or lost, or the SD card not be retrievable at the end of the survey. In the future, encrypting the data on the SD card would enhance data security, whereas storing data both on internal and external memory would further minimize risk of data loss. At completion of the survey, records were downloaded to local computers, staying in country without having to be transmitted over mobile phone networks or the Internet to a foreign server, maintaining sovereignty and physical possession of the data set by the host country.

There are alternatives to data storage, such as uploading to a cloud server via telecommunications networks or the Internet, or downloading from an internal disk on the device to a local computer. Each alternative has strengths and limitations. Methods for data collection must be designed to function within the limits of the local infrastructure and adherence to local guidelines on data management and security is mandatory. At the time our survey was conducted, the Internet connectivity infrastructure in Ethiopia has been given a score of 1 (thin) on a scale of 0 (non-existent) to 4 (immense) [39]. The software we used had the capability of web-based form design, deployment and data transfer, but our experience with connectivity during the pilot activities motivated us to pursue an off-line solution.

There were limitations in our study and these are offered for discussion. First, the comparisons made between data entry errors identified were from two different surveys implemented in different zones at different times of the year. Second, inherent to electronic data collection, there is little opportunity to confirm collected data by field teams simply by reviewing the data set as we did to identify errors. Yet, this same limitation applies to paper-based questionnaires; we assume that the recorded data were the actual response. Third, time to administer the survey as recorded in the pilot might be affected by factors other than the recorder’s ability to use the data collection tool, i.e., survey is interrupted by a neighbor or the respondent goes out to collect his/her children. We assumed that these external factors were, on average, the same for the two methods during the 2 days of observation, and hence do not affect the overall results. Fourth, the differences in mean distance to the cluster centroid should be interpreted with caution. Either households in South Wollo were simply more dispersed within community settlements than in South Gondar or the household distances from the cluster centroid were inflated due to systematic inaccuracies in transcribing household coordinates to paper in several clusters. The plots on Figure 3 and greater proportion of outlying households recorded in South Wollo suggest the latter. Finally, in estimating cost, we did not include the value of the time volunteered by the computer scientists (approximately 4 months of part-time work) to design and refine the system. Our reasoning was that free electronic data collection systems have now become available, although with less specific functionality, but could be deployed for survey use with only the added equipment costs and possibly training of survey coordinators on designing electronic forms and managing collected data. We also did not include the long-term value of the electronic equipment, which has continued to serve four other large-scale surveys in multiple countries at the time this manuscript was prepared for submission. Additional costs not considered were potential import duties levied on data collection devices and accessories as the regulations and amounts are setting specific, but should not be ignored when budgeting.

In summary, use of a novel system for electronic survey design, collection on an Android platform, and local management was feasible in a large-scale trachoma impact assessment survey. Electronic data collection saved time, was less costly, was at least as accurate as standard paper-based questionnaires, and was preferred by experienced paper-based data recorders. These advantages were similar as those advertised in recent applications of Android-based data collection applied to animal health and surveillance of zoonotic diseases [40], [41]. Such systems could be readily applied to other large-scale neglected tropical disease control surveys as well as national initiatives, such as the malaria indicator surveys (MIS), the demographic and health surveys (DHS), the UNICEF multiple indicator cluster survey (MICS), or the regular household surveys done by the health demographic surveillance systems (HDSS) of the INDEPTH network [42]–[44].

## Supporting Information

Table S1 **Description of hardware and software utilized for electronic data collection during the study activities.**
(DOCX)

Document S2 **Paper-based questionnaire as implemented in a large-scale trachoma survey in Ethiopia.**
(PDF)

Document S3 **Core questions for focus group discussions with data recorders from the pilot study team.**
(DOC)
