# Supplementary material for: A Novel Electronic Data Collection System for Large-Scale Surveys of Neglected Tropical Diseases
Source: PLoS One. 2013 Sep 16;8(9):e74570. doi: 10.1371/journal.pone.0074570 (PMC3774718; doi:10.1371/journal.pone.0074570)
Supplement: Table S1 — Description of hardware and software utilized for electronic data collection during the study activities. (DOCX) [file pone.0074570.s001.docx]

**Table S1.** Description of hardware and software utilized for electronic data collection during the study activities

| **Activity** | **Hardware** | **Software** |
| --- | --- | --- |
| *Pilot Study* Design electronic questionnaire | Desktop / Laptop computer | Build ODK: *browser application* Google Chrome browser |
| Data collection in field | Wistec A81E 7-in tablet computer  Blue-tooth external GPS | Android 2.0 Swift Insights Mobile 1.0 *Android App* |
| Data download and processing | Laptop computer | KoBoSync *Post* Processor : Java application for aggregating data |
| *Large-scale deployment* Design electronic questionnaire | Desktop / laptop computer | Swift Insights Desktop: *Java application* Mozilla Firefox browser |
| Data collection in field | Samsung Galaxy Tab GT-P1010 7-inch tablet computer | Android 2.1 Swift Insights Mobile 1.1 *Android App* Barcode scanner 4.3.1 *Android App* Amharic Android keyboard |
| Data download and processing | Laptop computer | Swift Insights Desktop: *Java application* |
