# Supplementary material for: A Novel Electronic Data Collection System for Large-Scale Surveys of Neglected Tropical Diseases
Source: PLoS One. 2013 Sep 16;8(9):e74570. doi: 10.1371/journal.pone.0074570 (PMC3774718; doi:10.1371/journal.pone.0074570)
Supplement: Document S2 — Paper-based questionnaire as implemented in a large-scale trachoma survey in Ethiopia. (PDF) [file pone.0074570.s002.pdf]

South Gondar Trachoma Impact Evaluation Survey – Amhara, Ethiopia: 2011

HOUSEHOLD QUESTIONNAIRE

Team number:	|___|___|	Cluster Number (1-380):	|___|___|___|	Household number:	|___|___|
Serial Number:	|___|___||___|___|___||___|___| Team Cluster Household		Gott name:		
Woreda name:			Health Cluster Name:		
Kebele name:			Development team name:		

Survey Date (DD/MM/YYYY) |___|___|/|___|___|/|___|___|___|___|	GPS	Latitude: (N) |___|___| **.** |___|___|___|___|___|___|
Household interview consent given? No= 0 Yes= 1		Longitude: (E) |___|___|___| **.**|___|___|___|___|___| ___|
Examination of children consent given? No= 0 Yes= 1		Elevation: |___|___|___|___|___| m

**Respondents' demographics (Adult females: women and mothers of children are the preferred respondents)**			
RD1	Name of head of Household	*Write name* ___________________________	
RD2	Description of Respondent	Head of household=1<br>Wife of head of household=2<br>Other	
RD3	Gender of Respondent	Male= M Female= F	
RD4	How old are you? *(round months down)*	*(write age in years)* |___| ___|	
RD5	What is the highest level of school you have attended? *(If "none," ask "have you had any non-formal education?")*	None=0<br>Religious=1<br>Primary school (grade 1-6)=2<br>Junior secondary=3<br>Senior secondary=4<br>College/University=5<br>Non-formal Education=6	
RD6	What is the HoH's primary occupation?	Farming and Cattle rearing = 1<br>Cattle rearing only = 2<br>Farming only = 3<br>Formal employment (monthly salary) = 4<br>Trade (business) = 5<br>Daily labourer = 6<br>Other *(write in)*________________ = 98	
RD7	Have you lived in this Gott for more than 4 years?	No=0; Yes=1	Yes go to ES1
RD8	If no, in which Woreda and Zone did you live recently?	(specify) _______________________________	
**Household Social Economic Status**			
	Does your household have any of the following?		
ES1	Functioning radio set	No=0; Yes=1	
ES2	Functioning Television	No=0; Yes=1	
ES3	Working electricity	No=0 ; Yes=1	
ES4	Functioning telephone (landline)	No=0 ; Yes=1	
ES5	Functioning mobile phone	No=0 ; Yes=1	
ES6	**Observation:** what is the main construction material for the roof in this household? *(One response only)*	Corrugated Iron=1<br>Thatch =2<br>Stick and mud=3<br>Other=99<br>(*specify*)_______________________________	
**Knowledge**			
K1	Have you ever seen anyone with this eye condition? *(link to show image TTwCO.jpeg)*	No=0; Yes=1	
K2	What is this condition called?	Trachoma=1<br>Cataract=2<br>I do not know=88<br>Other=99<br>(specify)_______________________________	
K3	Do you know what trachoma is?	No=0; Yes=1	If No, go to HE1

K4	In the end, what can happen to a person who has trachoma? *(multiple response)*<br><br>*(After each response ask 'anything else?' Do not read choices. Please mark all responses given.)*	K4.1 Nothing happens ☐<br>K4.2 Blindness ☐<br>K4.3 Reduced vision ☐<br>K4.88 I do not know ☐<br>K4.99 Other ☐<br>(*specify*)______________________________	
K5	How can someone protect him/herself from trachoma/trichiasis? *(multiple response)*<br><br>*(After each response ask 'anything else?' Do not read choices. Please mark all responses given.)*	K5.1 Face washing/hygiene ☐<br>K5.2 Take antibiotics or medicine ☐<br>K5.3 Trichiasis surgery ☐<br>K5.4 Keeping environment clean ☐<br>K5.5 Using pit latrines ☐<br>K5.88 I do not know ☐<br>K5.99 Other ☐<br>(*specify*)______________________________	
**Health Education**			
HE1	Have you ever heard health information on trachoma?	No=0 Yes=1	If No go to WS1
HE2	Where did you hear the trachoma information?<br><br>*(multiple response)*<br><br>*(After each response ask 'anything else?' Do not read choices. Please mark all responses given.)*	HE2.1 Trachoma volunteers ☐<br>HE2.2 Health extension worker ☐<br>HE2.3 Mass media (TV, radio, etc) ☐<br>HE2.4 Health facility ☐<br>HE2.5 Community gatherings ☐<br>HE2.6 School ☐<br>HE2.7 School child ☐<br>HE2.99 Other ☐<br>(*specify*)______________________________	
HE3	What information about trachoma did you hear?<br><br>*(multiple response)*<br><br>*(After each response ask 'anything else?' Do not read choices. Please mark all responses given.)*	HE3.1 Causes of trachoma ☐<br>HE3.2 Transmission of trachoma ☐<br>HE3.3 Latrine construction and use ☐<br>HE3.4 Face washing ☐<br>HE3.5 Antibiotics treatment ☐<br>HE3.6 Trichiasis surgery ☐<br>HE3.99 Other ☐<br>(*specify*)______________________________	
**Water source and access (Ask person responsible for water collection)**			
WS1	What is the main source of water your household uses for bathing?	Unprotected spring=1<br>Protected spring=2<br>Unprotected dug well=3<br>Hand pump/Tube well / borehole=4<br>Surface water (river, dam, lake, stream, canal)=5<br>Public piped water/ tap/standpipe=6<br>Private piped into Yard/dwelling=7<br>Rainwater collection=8<br>Other (*specify*)______________________=99	
WS2	What is the main source of water your household uses for washing clothes?	Unprotected spring=1<br>Protected spring=2<br>Unprotected dug well=3<br>Hand pump/Tube well / borehole=4<br>Surface water (river, dam, lake, stream, canal)=5<br>Public piped water/ tap/standpipe=6<br>Private piped into Yard/dwelling=7<br>Rainwater collection=8<br>Other (*specify*)______________________=99	

WS3	What is the main source of water your household uses for drinking?	Unprotected spring=1<br>Protected spring=2<br>Unprotected dug well=3<br>Hand pump/Tube well / borehole=4<br>Surface water (river, dam, lake, stream, canal)=5<br>Public piped water/ tap/standpipe=6<br>Private piped into Yard/dwelling=7<br>Rainwater collection=8<br>Other (*specify*)______________________=99	
WS4	How long does a round trip take to collect water from the source of water used for **bathing**?	<30 minutes=1<br>30 minutes to 1 hour=2<br>> 1 hour=3	
**Face washing (Ask mothers or care givers of children)**			
FW1	Do you have children under 5 years of age?	No=0; Yes=1	If No, go to PL1
FW2	How often are these children bathed? *(Indicate one answer)*	Never=0<br>Every other day=1<br>Once a day=2<br>Twice a day=3<br>Three or more times a day=4<br>Other (*specify*)______________________=99	
FW3	How often are the faces of children washed? *(Indicate one answer)*	Never=0<br>Every other day=1<br>Once a day=2<br>Twice a day=3<br>Three or more times a day=4<br>Other (*specify*)______________________=99	
**Pit Latrines**			
PL1	Have you ever had a latrine in this household?	No=0; Yes=1	If No go to LO2 (note there may still be a hand-washing container in the HH)
PL2	Currently, is there a latrine in this household (observed)?	No=0; Yes=1	
PL3	Is this the first latrine in this household?	No=0; Yes=1	
PL4	How long ago was the most recent latrine built?	_________***YEARS*** ________***MONTHS ago***	
**Latrine observations**			
LO1	Evidence of latrine usage (*faeces in pit)?*	No=0; Yes=1	
LO2	Hand washing container present?	No=0; Yes=1	If no to LO2, END
LO3	Water in hand washing container?	No=0; Yes=1	
LO4	Location of hand washing container?	At latrine=1; At household=2	

Serial Number (same as HH quest.) |___|___|___|___|___|___|___| HH #: |___|___| Cluster #: |___|___|___|

					Absent	School	Antibiotic treatment during MalTra (tablets, POS or TEO)				Use latrine	Discharge No=0; Yes=1		Right Eye No=0; Yes=1					Left Eye No=0; Yes=1					If TT	PCR or STH
ID. #		Name	Sex M/F	Age	Present=0 Out=1 Travel=2 No consent=3 At school=4	No=0 Yes=1	Ever taken No=0 Yes=1	# times taken	In register? No=0 Yes=1	Response Verified No=0 Yes=1	Always=1 Some=2 Never=3	Ocular	Nasal	TT	CO	TF	TI	TS	TT	CO	TF	TI	TS	Write # lashes on globe	Write PCR, STH, or both if selected
0	1																								
0	2																								
0	3																								
0	4																								
0	5																								
0	6																								
0	7																								
0	8																								
0	9																								
1	0																								
