# Supplementary material for: A Novel Electronic Data Collection System for Large-Scale Surveys of Neglected Tropical Diseases
Source: PLoS One. 2013 Sep 16;8(9):e74570. doi: 10.1371/journal.pone.0074570 (PMC3774718; doi:10.1371/journal.pone.0074570)
Supplement: Document S3 — Core questions for focus group discussions with data recorders from the pilot study team. (DOC) [file pone.0074570.s003.doc]

**Document S2. Questions to prompt group discussion concerning use of electronic and paper questionnaires**

After using both paper and electronic questionnaire:

1. What are the advantages and disadvantages of each method?

2. Which data collection method do you prefer and for what reasons?

3. What problems did you have when collecting data using either method?

4. Which data collection method do you think took less time to complete during the survey and why?

5. With which tool do you think an interviewer will make fewer mistakes and why?

6. How did you feel about using the tablet computer to collect data?

7. What would you suggest adding or changing in order to make use of the tablet computers easier?

8. Which sections of the electronic questionnaire did you find difficult to use?

9. In what areas of electronic data collection do you feel you need more training?
